# Frontline Service Employees’ Profiles: Exploring Individual Differences in Perceptions of and Reactions to Workplace Incivility

**DOI:** 10.3390/bs12030076

**Published:** 2022-03-10

**Authors:** Boshra H. Namin, Einar Marnburg, Åse Helene Bakkevig Dagsland

**Affiliations:** Norwegian School of Hotel Management, University of Stavanger, 4021 Stavanger, Norway; einar.marnburg@uis.no (E.M.); aase-helene.b.dagsland@uis.no (Å.H.B.D.)

**Keywords:** workplace incivility, turnover intention, social supports at work, frontline service employees, cluster analysis

## Abstract

Employee turnover is a big issue in the service industry, which can be significantly affected by job stressors including workplace incivility. This exploratory study aims to identify the frontline service employees’ profiles exploring to what extent individuals may have different perceptions of incivility and social supports at work and showing different reactions (job outcomes). In a cross-sectional study, 291 completed questionnaires from a sample of Norwegian frontline service employees were subjected to correlation analysis, K-means clustering, and post hoc ANOVA analysis with Bonferroni correction. Cluster analysis revealed three distinct clusters of employees with different profiles, which indicated that those who perceived the highest level of workplace incivility and the lowest level of social supports at work showed the highest turnover intention compared to that of others. Moreover, employees with longer tenure and the highest perception of social supports at work coped better with workplace incivility and showed the lowest turnover intention.

## 1. Introduction

The “turnover issue” in tourism and hospitality has received enormous research interest in the last decade. The reason is obvious: tourism, hotel, and restaurant sectors report very high turnover figures (e.g., [1,2,3]). Although the significant role of frontline service employees is confirmed by both managers and scholars, research has consistently indicated that service employees are generally untrained, overworked, and highly stressed [4]. Not surprisingly, therefore, their turnover intentions have been asserted as one of the important and continuous challenges for service managers [1,5], since high turnover gives high recruiting costs, problems with a stable level of service delivery, and may have a negative influence on the work environment. Researchers have studied various factors influencing turnover, for example, external job alternatives, work–life balance, employment conditions, and the work environment [6]. Studies into the work environment are especially interesting because the work environment may be influenced not only by the industries and local management that are taking active roles in HR systems, but also by how jobs are organized, what culture employees experience, and how well managers do their jobs.

Frontline service employees have to deal with multiple interpersonal stressors from different sources in the work environment [2]. A stressful situation can be created when these employees experience negative social interactions with customers and coworkers [7]. As a very widespread phenomenon in the work environment, workplace incivility is the major factor in job stress [8]. A research track has studied occurrences and effects of uncivil behaviors from coworkers and customers on frontline employees (e.g., [9,10]). Research has identified workplace incivility from customers and coworkers as a negative stressor in work environments for frontline employees, which may lead to emotional exhaustion and, in turn, negative job outcomes such as low performance [11] and high turnover [12,13,14]. Some managerial decisions and actions may significantly improve the work environment for the employees. According to [15], with the perception of social support(s) in the organization, employees will have a significant emotional resource, which helps them to control their emotions more effectively and allows them to cope well with job stressors. Research within this track has also demonstrated how a caring organizational climate and a good relationship between frontline employees and their managers can reduce the negative effects of incivility [16,17]. However, most of this research holds a functional perspective of how organizations work in forming employees’ attitudes and behaviors by considering people as a product of social groups and organizational environment. From such a perspective, problems are solved by organizational policies, HR management, or local management. Individual differences in employees’ responses to organizational actions and their coping behavior are not given much attention. Although some employees respond very well to managerial actions and support at work, there could be some other employees who do not. This may arise a question about the role of individual differences in employees’ perceptions and reactions to negative interpersonal interactions in the workplace.

In this exploratory paper, we explored differences in individual behaviors and reactions against the perception of workplace incivility among a sample of frontline employees in the hotel and restaurant industry. Our main question was whether it was possible to identify groups of employees that behave differently from other groups. If so, then could we recognize a behavioral pattern and demographic profile of such groups of individual employees? We believe that such insights into individual differences in (a) how employees perceive and cope with stressors and (b) to what extent the organization and managers affect their behaviors will be of great interest for both research and practice. Individual differences explain the variance when measuring the effects of, for example, the mediating role of managements’ effort to reduce employees’ turnover.

In the next section, previous research-based knowledge of incivility behavioral effects will be elaborated with a focus on studies that identify individual differences among frontline service employees.

## 2. Background Literature

### 2.1. Workplace Incivility and Job Outcomes

Workplace incivility refers to “low-intensity deviant behavior with ambiguous intent to harm the target, in violation of workplace norms for mutual respect. Uncivil behaviors are characteristically rude and discourteous, displaying a lack of regard for others” [18] (p. 457). Examples of workplace uncivil behaviors include one’s negligence in saying “please” or “thanks”, using snippy voice, disrupting meetings, verbal attacks, withholding important information, spreading rumors, taking credit for other’s efforts, and leaving rude messages [19,20]. Perception of incivility in the organization affects employees by different negative behavioral, physiological, and psychological outcomes [21]. As a consequence of the specific attributes of service jobs including deep-rooted stress [9], reliance on coworkers [7], and the close connection between frontline employees’ performance and customer encounters in the service industry [10], these employees are specifically targeted to incivility from two main internal and external sources (i.e., coworker and customer) in their daily working life.

According to previous research (e.g., [9,13,22,23]) workplace incivility perception among service employees has damaging effects on individual and organizational outcomes. Sustained exposure to workplace incivility results in emotional exhaustion [24], which in turn, decreases employees’ job performance [11,23] and increases their turnover intention [25]. The positive association between incivility and absenteeism, lateness, and job-quitting behavior as well as its antecedent role in the formation of turnover intention has been identified in previous research (e.g., [2,14,26,27]).

### 2.2. The Role of Individual Characteristics and Personality Traits

In an investigation of how individual characteristics (i.e., age, education, job tenure, and experience) affect service employees’ job-related responses, [28] indicated that the employees who are older, better educated, and have higher service experience tend to be more satisfied and committed while coping better with job stressors, which may result in lower absenteeism and turnover. With a focus on Big Five factors and the Dark Triad of personality as individual differences, [29] demonstrated that they are potential predictors of uncivil meeting behaviors including inappropriate interpersonal behavior (e.g., offending others) and dominant communication behavior (e.g., interrupting others). Moreover, [30] showed a double-edged effect of narcissism, that is, narcissistic employees reacted to workplace incivility with higher levels of anger on the one hand that results in lower family satisfaction. On the other hand, they were less likely to feel guilty when experienced incivility and thus, maintained family satisfaction. According to [30], family satisfaction is an indicator of non-professional well-being characterized by individuals’ satisfaction level with their family relationships [31].

Another study about individual differences based on personality traits showed that employees who are high in neuroticism and low in agreeableness experience more incivility in the workplace [32]. In a recent study, the role of individual differences in generating specific emotions and behaviors in response to workplace incivility was investigated [33]. The result showed that the employees with higher internal attribution orientation can care more about others’ feelings and take more responsibility for unpleasant interactions. Thus, they are more likely to feel guilty when they perceive incivility from coworkers, and to reduce this feeling, they show more positive behaviors.

### 2.3. Workplace Social Supports

The role of service managers is particularly notable in creating a more favorable work environment for the employees. Leader-member exchange (LMX) quality [34] is an important social support in the organization, which emphasizes the leaders and followers’ dyadic relationship. It refers to the employees’ perception of the quality of their interpersonal social exchange with their managers [35]. In high-quality LMX, managers may provide social support for the employees by showing empathy and support for their needs [36]. Employees with a high perception of LMX quality are more likely to accomplish challenging tasks [37] and show higher performance [38,39] and lower turnover intention [40]. The result of a recent time-lagged study demonstrated that employees with low-quality LMX relationships with the manager instigated more uncivil behaviors toward their coworkers [41]. Previous studies also revealed damaging effects of high LMX differentiation on group performance and employees’ relationships [42,43].

Moreover, the caring climate has been considered as a significant factor to address the association between job stressors and related job outcomes [17]. A caring climate refers to shared perceptions of organizational policies, procedures, and systems among the employees that affect their behaviors by emphasizing friendship and team interest [44]. Through establishing a caring climate, managers would be able to develop positive attitudes among employees, motivate them to consider the effect of their behaviors, lead them to make decisions according to the interest of others’ well-being [16], and feel obliged to help other members and coworkers [45]. In such an atmosphere, even by experiencing a high level of stressors, the employees are more likely to show positive job outcomes [17] through higher job performance [46] and lower intention to quit their jobs [26].

A theory that can provide a foundation for a deeper understanding of the relationship between workplace incivility and its impacts on different job outcomes is the conservation of resource (COR) theory [47]. Resources are an essential part of COR theory and refer to any valuable objects, personal characteristics, conditions, or energies that an individual uses to attain these resources again [47]. The main issue is the limitation of such priceless resources, and that is why people seek to achieve, maintain, conserve, and foster them [47,48] to cope with stressors. However, these resources could be gradually lost while, for example, someone needs to deal with workplace incivility. In the dark tenets of COR theory, employees may try to restore loss of resources by reducing their performance [49] and showing withdrawal behaviors [7] including turnover intention. Based on this theory, frontline service employees who are exposed to incivility from customers and coworkers in their daily working life (resource drain), experience more stress and emotional exhaustion and finally seek to restore their resources [47]. That is, the emotionally exhausted employees may show negative reactions that could adversely affect their job outcomes. In this study, we considered the employees’ perception of a caring climate and LMX quality as two supplementary resources and social supports at work.

## 3. Methodology

### 3.1. Sampling and Procedure

A cross-sectional design with purposive sampling was applied for this study. Purposive sampling is used as a nonrandom sampling technique depending on the deliberate choices and judgments of the researcher about selecting a sample of individuals who agree to voluntarily provide required information based on their knowledge and experience [50]. The questionnaires of the present study were distributed among frontline service employees who work as receptionists, waiters or waitresses, or bartenders in several hotels and restaurants in Norway. Frontline service employees have been selected for this study because of their frequent face-to-face or voice-to-voice interactions with the customers/guests that imply their key role in building loyalty and improving customers’ pleasure through managing their requests and resolving their problems [51]. Moreover, being heavily reliant on coworkers [7] and having deep-rooted stress [9] in providing high-quality service for the customers as the specific features of service jobs expose these employees to workplace incivility more than any other staff in the hotel and restaurant industry.

### 3.2. Data Collection

The data was collected from undergraduate students in tourism/hotel management at a university in Norway. Only the students who had been working for at least six months in a hotel or a restaurant before participation in this study were eligible to complete the self-administrated questionnaire. The questionnaires were in English. The understandability of the questionnaire items was checked in a pre-test among 10 Master’s students in the same field, and the necessary changes were made accordingly. On the first page of the questionnaire, the aim of the study, contact information, the voluntary nature of participation, and full anonymity and confidentiality were emphasized. Participants needed approximately 10–15 min to fill out the questionnaire.

As highlighted in previous studies (e.g., [52,53]), to decrease the potential threat of common method bias (CMB), a special box was provided to keep the completed questionnaires. One of the researchers obtained this box after data collection. A total of 465 questionnaires were distributed and 322 were returned, resulting in a 69.2 percent response rate. The questionnaires with more than 20 percent unanswered items were considered as missing data and after their deletion, 291 responses were used for data analysis.

### 3.3. Measures

The Perception of customer incivility was measured through 4 items adapted from [54], who have validated the Incivility from Customer Scale (IFCS). One of the included items was “How often have customers blamed you for a problem you did not cause?”. The perception of coworker incivility was measured through 4 items adapted from [55], who have developed and validated the Uncivil Workplace Behavior Questionnaire (UWBQ). “How often have your coworkers made unkind/mean remarks about you in a clever indirect way?” was among the included items. The 5-point Likert scale measure ranging from 1 (never) to 5 (very often) was used for these two variables.

The perception of a caring climate was measured through 4 items from [44]. One of the included items was “The managers are very concerned about what is generally best for the employees in this workplace”. To measure LMX quality, 3 items were adapted from [56]. “I characterize my working relationship with my supervisor as very effective” was one of the included items. Emotional exhaustion was measured through 3 items from the Maslach Burnout Inventory [57]. One of the included items was “I feel used up at the end of the workday”. Job performance was measured through 3 items adapted from [58]. “I am a top performer” was among the included items. The turnover intention was measured through 3 items from [59]. One of the included items was “I will accept a contract offer from another organization if it comes tomorrow”. The 5-point Likert scale ranging from 1 (strongly disagree) to 5 (strongly agree) was used for all of these variables.

For personal characteristics and working conditions, respondents’ gender (male = 0, female = 1), tenure (6–11 months = 1, 1–3 years = 2, 4–5 years = 3, and more than 5 years = 4), industry (hotel = 1 or restaurant = 2), and supervising responsibility (dichotomous response of yes = 1 or no = 2) were considered in this study. Table 1 shows the demographic profiles of the respondents. The majority of the participants were female (66%), worked at hotels (65%), with 1–3 years of experience (45%), and did not have a supervising position (75%).

### 3.4. Data Analysis

The collected data were analyzed with Statistical Package for Social Science (IBM SPSS) version 26. Descriptive statistics, reliability tests, correlation analysis, K-means cluster analysis, and one-way ANOVA were conducted. Cluster analysis aims to group the participants who similarly responded to the questions and explore the considerable heterogeneity in their characteristics [60]. Accordingly, K-means cluster analysis [61] was performed to find out a structured view of the participants. The advantage of a K-means clustering is to enable cases to be relocated to new clusters in an iterative process, so their locations would be potentially improved with no changes in the specific number of clusters during iterations. We checked different theoretically relevant numbers of clusters in several runs, and it revealed that a solution with three distinct clusters was the most informative. Finally, one-way ANOVA was performed to discriminate between the clusters using post hoc Tukey’s pairwise comparisons.

## 4. Results

Table 2 presents the correlation coefficient between all included variables in the present study. Significant positive correlations were found between workplace incivility and emotional exhaustion (0.28 for both customer and coworker incivility). However, customer incivility did not show a significant correlation with both turnover intention (0.08) and job performance (0.10), while coworker incivility showed a significant correlation with turnover intention (0.17). Emotional exhaustion had a positive and significant correlation with turnover intention (0.52), but a positive and insignificant correlation with job performance (0.06). Both customer (−0.13) and coworker incivility (−0.25) had a significant negative correlation with caring climate, but only coworker incivility had the same correlation with LMX quality (−0.16). Correlations between social supports and emotional exhaustion were negative and significant (−0.39 for LMX quality and −0.36 for caring climate). They also showed significant negative correlations with turnover intention (−0.49 for LMX quality and −0.45 for caring climate). However, only LMX quality showed a significant correlation with job performance (0.28).

The reliability of the variables was checked through Cronbach’s alpha test, which showed 0.76 for customer incivility, 0.70 for coworker incivility, 0.82 for emotional exhaustion, 0.73 for job performance, 0.73 for turnover intention, 0.85 for LMX quality, and 0.89 for the caring climate. In line with [61], the reliability of our constructs was satisfactory since all Cronbach’s alpha scores were higher than 0.60. 

**Figure 1:**
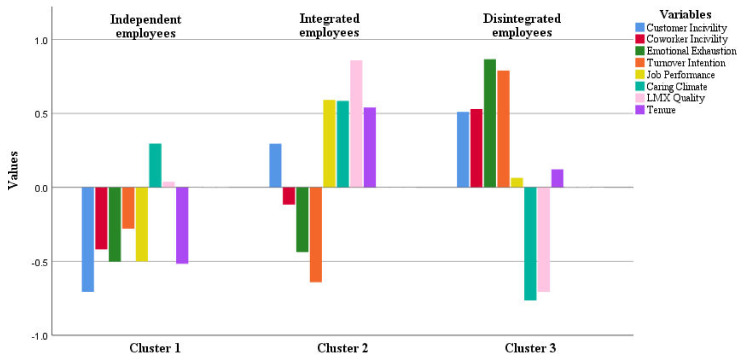
Final cluster solution for frontline service employees’ profiles (three-cluster solution).

Prior to performing K-means cluster analysis, the numbers of final groups (clusters) should be decided. Considering the exploratory nature of cluster analysis, we analyzed several solutions in this study. After evaluating the validity coefficient Kappa for two-to five-cluster solutions, the three-cluster solution was identified as the best solution with the highest value compared to two, four, and five clusters. In line with the purpose of this study to find different groups of employees with clear distinction, the three-cluster solution could achieve the most informative and meaningful results. The three profiles of frontline service employees that emerged from the final cluster solution were designated based on employees’ perception of workplace incivility and social supports at work as well as their job outcomes. The clusters were labeled as “Independent employees”, “Integrated employees”, and “Disintegrated employees”, respectively. These terms (i.e., Independent, Integrated, and Disintegrated) have been selected based on general behavioristic and demographic differences among three clusters considering employees’ profile characteristics, final inference from their behaviors, and the relationships established at work. A detailed description of these differences is presented in the next subsections for each cluster. These three clusters are clearly illustrated in Figure 1, and their initial comparison is available in Table 3. The ordinal ratios in Table 3 (i.e., low, medium, and high) are based on grouping the values into three groups by reducing the medium and defining three score range brackets (33.33% in each group).

### 4.1. Independent Employees

Table 4 and Table 5, respectively, present the demographic and behavioral profiles of the three clusters and their comparison. Based on the results, the majority of the employees in cluster 1 were female (59%) and worked at hotels (56%). Most of them did not have a supervising position (91%), which is the highest rate compared to those in other clusters. Moreover, they had the lowest tenure, and 85% of them had less than three years of work experience. 

Regarding the perception of workplace incivility, the results demonstrated that Independent employees perceived both customer and coworker incivility at the lowest rate compared to the other clusters. They perceived a lower level of customer incivility than coworker incivility, though. They also felt the lowest emotional exhaustion. Their perception of social supports at work was not very high, however, they perceived a caring climate much more than LMX quality. Finally, they showed weak job outcomes since they had the lowest job performance, but their turnover intention was low too. 

### 4.2. Integrated Employees

In the second cluster, 61% were female, 86% worked at hotels (the highest rate compared to those of other clusters), and 55% did not have a supervising position. More than half of them (51%) had more than four years of work experience, which was the highest tenure rate compared to those of other clusters.

Based on the results, these Integrated employees perceived a high level of customer incivility but a lower level of coworker incivility at the workplace and felt low emotional exhaustion. They also had the highest rate in the perception of social supports at work in both caring climate and LMX quality among the clusters. They perceived LMX quality at a higher level than caring climate, though. Eventually, they demonstrated the best job outcomes, since, compared to the other clusters, they had the highest job performance and the lowest turnover intention at the same time.

### 4.3. Disintegrated Employees

Like the other two clusters, the majority of employees in cluster 3 (78%) were female (also the highest rate compared to the other clusters), worked at hotels (59%), and did not have a supervising position. Only 35% of them had more than four years of work experience.

The results showed that Disintegrated employees had the highest perception of workplace incivility compared to the other clusters, as they perceived both customer and coworker incivility relatively at the same level. They also felt the highest emotional exhaustion. In addition, they perceived the lowest social supports at work among the clusters, where their perception of a caring climate was lower than LMX quality. Consequently, they showed the weakest job outcomes since they had low job performance and the highest turnover intention rate compared to those of the other two clusters.

We performed a chi-squared test and post hoc ANOVA analyses, using Bonferroni correction [62,63] to investigate any significant differences in demographic characteristics and working conditions between the three clusters (Table 4), as well as using Tukey’s test to check any significant mean differences for behavioral variables between the clusters (Table 5). The chi-square test for gender, tenure, industry, and supervising position of the respondents was statistically significant. There was no significant mean difference for the perception of customer incivility between Integrated and Disintegrated employees (cluster 2 and 3, *p* = 0.20). Independent and Integrated employees (clusters 1 and 2) did not have significant mean differences for the perception of coworker incivility (*p* = 0.06) and emotional exhaustion (*p* = 0.83). More details of the chi-square, ANOVA, and post hoc analyses are demonstrated in Table 4 and Table 5. 

## 5. Discussion

In line with the aim of this explorative study, we explored the demographic and behavioral profiles of frontline service employees concerning their perception of workplace incivility and social supports at work and their relevant job outcomes in the hotel and restaurant industry. We identified three distinct clusters: (1) Independent employees, (2) Integrated employees, and (3) Disintegrated employees. Independent employees had a low perception of workplace incivility and a mid-range perception of social supports. They experienced low emotional exhaustion, and their very low job performance and low turnover intention led us to conclude that they showed weak job outcomes in general. Integrated employees showed a mid-range perception of workplace incivility and a high perception of social supports. They experienced low emotional exhaustion, and their job performance was very high while their turnover intention was very low, which implies highly satisfactory job outcomes of Integrated employees. Disintegrated employees, on the other hand, had a high perception of workplace incivility and a low perception of social supports. With a high level of emotional exhaustion, lower job performance, and a very high level of turnover intention, they demonstrated the weakest job outcomes.

This study confirmed previous research findings of the positive association between workplace incivility from both internal and external sources (i.e., coworkers and customers), emotional exhaustion, and turnover intention [2,11]. It also confirmed the negative association between both sources of social support (i.e., LMX quality and caring climate) and employees’ turnover intention [26,64]. Interestingly, job performance was not found to have any negative correlations with workplace incivility (0.10 for customer and 0.001 for coworker incivility) and emotional exhaustion (0.06). The rational explanation behind this result could be in the classification of stressors into hindrance and challenging stressors [65]. Although hindrance stressors are perceived by employees as barriers that threaten their personal growth and accomplishment of goals with detrimental effects on their job outcomes, employees perceive challenging stressors as work-related demands or encounters that are favorable and beneficial to support their goals [65,66]. Challenging stressors have a positive effect on employees’ job outcomes, increasing their positive work attitudes [65,66]. Accordingly, it seems that participant employees in this study perceived workplace incivility as a challenging stressor at some level, which, in turn, did not negatively affect their job performance.

Interpersonal interactions and stressful situations at work did not significantly affect Independent employees, and therefore, they did not become emotionally exhausted. However, despite their low level of job performance, they were relatively less likely to leave their job. This is inconsistent with the previous result showing a negative relation between job performance and turnover intention (e.g., [67]). A low level of turnover intention among Independent employees could be due to their perception of social supports at work. Specifically, their perception of a caring climate was quite good. Working in a decent environment with a caring orientation and perception of a relatively good relationship with the managers (LMX quality) could lead Independent employees to stay in the organization [17,26]. On the other hand, their low tenure and the fact that most of them (85%) had less than three years of work experience (one half with 6–11 months and the other half with 1–3 years) may lead them to stay in their current positions since it could be more difficult for them (than long-tenured employees) to find another job in the near future.

In contrast to the previous evidence about the negative effect of workplace incivility on employees’ psychological outcome and emotional exhaustion (e.g., [10,13,21]), Integrated employees in this study experienced low emotional exhaustion, although they perceived a high level of customer incivility. This was strongly due to their high perception of social supports. They perceived even higher LMX quality than caring climate, which represented the strong relationships they had with their managers compared to other employees. Not surprisingly, Integrated employees did not show negative reactions to stressful situations (i.e., workplace incivility) and demonstrated a low level of turnover intention, which is inconsistent with the previous result showing a positive relationship between workplace incivility and turnover intention (e.g., [2,68]). The reason behind this finding could be again in their high perception of social supports. There is strong evidence that perception of LMX quality is negatively related to turnover intention [3,69] and positively related to job performance [70]. The caring climate is also identified to have an antecedent role in reducing turnover intention [64] and increasing job performance [46]. Moreover, Integrated employees had the highest tenure rate compared to others, and more than half of them (51%) were long-tenured employees (22% had 4–5 years, and 29% had more than 5 years of experience). This is in line with previous research (e.g., [28,71]), which demonstrated the relationship between employees’ higher tenure and lower turnover intention.

The high level of emotional exhaustion among Disintegrated employees could be explained by COR theory, since dealing with negative interpersonal interactions at work (i.e., incivility from customers and coworkers) drains emotional resources that employees need for using later [24]. As an effort to restore valuable resources, Disintegrated employees showed very negative reactions through reducing their job performance and showing a very high level of turnover intention. This is in line with previous studies that applied COR theory and demonstrated the negative effect of workplace incivility on employees’ emotional exhaustion, lower job performance [23], and higher turnover intention [25]. In addition to the short tenure of Disintegrated employees (only 35% had more than 4 years of work experience), their very low perception of social supports (in both LMX quality and caring climate) could be another reason for their negative job outcomes. When the employees’ perception of their relationships with managers is negative, they could become demotivated and dissatisfied, not feel obliged to work harder, and in turn, show lower performance [38,39] as well as higher turnover intention [40]. A lack of support and not being considered in the organizational decision-making process may lead Disintegrated employees to feel that the management and the organization do not care about their well-being. Thus, based on the results in this study, the low perception of a caring climate negatively affected their performance [46] and increased their turnover intention [17,64].

## 6. Knowledge and Research Implications

This study was an attempt to explore to what extent frontline service employees who work in similar working environments, may have different perceptions and behaviors, which could enable us to categorize them in distinct groups with specific profiles. Although workplace incivility and its related job outcomes including turnover intention have attracted scholars’ attention in previous research, still little is known about the role of individual demographic and behavioral differences in explaining their perceptions and responses to negative interpersonal interactions in the workplace. Our analyses indicated that employees were not only dissimilar in perceiving interpersonal interactions at work, but they also perceived the working environment and managerial actions very differently, which resulted in their different job outcomes. Therefore, this study advances the understanding of workplace incivility research by showing that individual differences merit careful attention in organizational practice and future research. With emphasizing the role of valuable emotional resources, this study lends further support to COR theory [49] and illustrates that perception of social supports at work can compensate for the resource depletion resulting from dealing with workplace incivility. Integrated employees versus Disintegrated employees in the current study is a good example of such a process. Independent employees also showed that even a lower level of perception of social supports led to lower turnover intention.

### 6.1. Practical Implications

The findings of the current paper provide new insight into the service managers’ awareness of the employees’ individual differences in perception of the same interpersonal interactions and working environment. This should be carefully noticed by the service managers at the time of providing a well-designed organizational structure, effective management intervention, and supporting improvements in frontline employees’ working life to prevent negative job outcomes and decrease turnover rate. Here, we offer a number of practical suggestions for our distinct clusters. 

The second cluster in this study (Integrated employees) was the *premier* group, which included the best employees from the service managers’ perspective due to their best job outcomes compared to other clusters. Service managers are extremely required to keep them motivated and involved in the organization through, for example, effective communication and feedback process as well as using staff retention strategies. Training and preparation for effectively coping with customer incivility are useful [13]. Obtaining detailed information about customer incivility incidents and employees’ aftermath feelings could also help the managers to understand the magnitude of the damage and develop a response strategy to diminish the negative effects [12].

The first cluster (Independent employees) was the *potential* group, which included employees who perceived low social supports while showing low turnover intention. As a big advantage for the service managers, these employees had the potential for improvement to get into the second cluster and become Integrated. Managers may provide ongoing education with practical guidance for them to manage their emotions at work. Managers can also provide clear career paths and explicit job descriptions, promote healthy interpersonal interactions, establish an effective manager–employee relationship, and develop friendship and team interest to encourage positive behaviors [17,38]. All of these would help Independent employees to feel cared for and secured in the job positions, which may, in turn, increase their performance and keep them longer in the organization [46,64] since according to our results, longer-tenured employees are more Integrated.

The third cluster that included Disintegrated employees was the *problematic* group with the highest negative perceptions and the highest level of turnover intention. Dealing with such employees requires a tremendous effort. Managers should cultivate positive emotions that provide personal resources for these employees through HR practices including training programs, workshops, and arranging personal growth and competence to intrinsically motivate these employees [72]. Perhaps, focusing on an appropriate recruitment process could be more effective, which can be done by identifying, attracting, hiring, and retaining intrinsically motivated staff from the beginning [72]. Some selection approaches, such as asking for employees’ referrals and clarifying expectations in advance, could be helpful here. 

### 6.2. Limitation and Future Research

Despite interesting results, this study still has its limitations. The cross-sectional design of this study imposes a potential limitation of causal inferences since it does not lead to conclusions about causal directions of the relationships. It also cannot identify the factors that lead to the gradual movement of an individual from a cluster to another one during a long period. These issues can only be explored with longitudinal design in future studies. The generalization of our results might be limited due to the sample of 291 frontline service employees working in the hotel and restaurant industry in Norway. Future studies may consider other frontline service employees such as airline cabin crews in Norway or the same employees in other countries to investigate if there is the same pattern in profiles of the employees’ clusters. In future research, supervisor incivility could be also included to provide more comprehensive information about the effect of the perception of workplace incivility on turnover intention and to investigate the contradiction between the positive and negative sides of the managerial role in the service industry. While cluster analysis has the strong ability to generate meaningful subgroups in data, the characteristics of these subgroups were strongly affected by the choice of variables. This can be considered as a limitation, since a clear theoretical underpinning was not available to show the best way of selecting variables for subject classification. Thus, different experimental studies should be conducted to explore the role of individual differences in perceptions and responses in a service context. It is also interesting to go to the other side of workplace incivility and explore if it is possible to identify different groups of *instigators* with different demographic and behavioral profiles.

## Figures and Tables

**Table 1:** Demographic profiles of the respondents (*n* = 291).

Variable	Category	Frequency (*n* = 291)	Percentage (100%)
Gender	Male	98	33.7
	Female	193	66.3
Tenure	6–11 months	65	22.3
	1–3 years	132	45.4
	4–5 years	50	17.2
	More than 5 years	44	15.1
Industry	Hotel	190	65.3
	Restaurant	101	34.7
Supervising position	Yes	74	25.4
	No	217	74.6

**Table 2:** Pearson correlation coefficients between the study variables (*n* = 291).

Variables	1	2	3	4	5	6	7	8	9	10	11
1. Customer incivility	-										
2. Coworker incivility	0.29 **	-									
3. Emotional exhaustion	0.28 **	0.28 **	-								
4. Job performance	0.10	0.001	0.06	-							
5. Turnover intention	0.08	0.17 **	0.52 **	−0.02	-						
6. LMX quality	−0.09	−0.16 **	−0.39 **	0.28 **	−0.49 **	-					
7. Caring climate	−0.13 *	−0.25 **	−0.36 **	0.06	−0.45 **	0.53 **	-				
8. Gender	0.05	−0.09	0.13 *	0.04	0.05	−0.12 *	−0.12 *	-			
9. Tenure	0.19 **	0.11	0.06	0.14 *	−0.002	−0.01	−0.07	0.06	-		
10. Industry	−0.07	0.07	0.02	−0.02	0.08	−0.07	−0.17 **	0.08	0.03	-	
11. Supervising position	−0.06	−0.13 *	−0.05	−0.24 **	−0.09	−0.07	0.05	0.03	−0.25 **	−0.04	-

Notes: ** Correlation is significant at the 0.01 level (two-tailed), * correlation is significant at the 0.05 level (two-tailed).

**Table 3:** Initial comparison of three clusters.

Variables	Clusters		
	(1) Independent Employees	(2) Integrated Employees	(3) Disintegrated Employees
Customer incivility	Low	Medium	High
Coworker incivility	Low	Medium	High
Emotional exhaustion	Low	Medium	High
Job performance	Low	High	Medium
Turnover intention	Medium	Low	High
LMX quality	Medium	High	Low
Caring climate	Medium	High	Low

Notes: Low < 33.33%; 33.33% < Medium < 66.66%; High > 66.66%.

**Table 4:** Demographic profiles of the three clusters and their comparison.

Personal Characteristics and Working Conditions	Clusters						Overall (*n* = 291)			Between Clusters		
	(1) Independent Employees (*n* = 108)		(2) Integrated Employees (*n* = 80)		(3) Disintegrated Employees (*n* = 103)					Post Hoc Tests (Bonferroni Correction)		
										(1)	(2)	(3)
	*n*	%	*n*	%	*n*	%	Mean (SD)	χ^2^	*p*	*p*	*p*	*p*
Gender (M/SD)	(0.59/0.49)		(0.61/0.49)		(0.78/0.42)		0.66 (0.47)	16.95	***	***	0.81	***
Male	44	40.70	31	38.75	23	22.30						
Female	64	59.30	49	61.25	80	77.70						
Tenure	(1.75/0.77)		(2.78/0.90)		(2.37/0.96)		2.25 (0.97)	44.26	***			
6–11 months	46	42.60	2	2.50	17	16.50				***	***	0.58
1–3 years	46	42.60	37	46.25	49	47.60				0.63	0.18	0.33
4–5 years	13	12.00	18	22.50	19	18.45				0.18	0.35	0.74
More than 5 years	3	2.80	23	28.75	18	17.50				***	***	0.71
Industry	(1.44/0.50)		(1.14/0.35)		(1.41/0.49)		1.35 (0.48)	9.00	**	**	0.49	**
Hotel	60	55.60	69	86.25	61	59.20						
Restaurant	48	44.40	11	13.75	42	40.80						
Supervising position	(1.91/0.29)		(1.55/0.50)		(1.73/0.45)		1.75 (0.44)	100.20	***	***	***	0.10
Yes	10	9.25	36	45.00	28	27.20						
No	98	90.75	44	55.00	75	72.80						

Notes: *** *p* < 0.001, ** *p* < 0.05, *p* from post hoc ANOVA for mean comparison (two-tailed) and from Chi square test for percentage comparison.

**Table 5:** Behavioral profiles of the three clusters and their comparison.

Main Variables	Clusters						Overall (*n* = 291)	Between Clusters		
	(1) Independent Employees (*n* = 108)		(2) Integrated Employees (*n* = 80)		(3) Disintegrated Employees (*n* = 103)			Post Hoc Tests (Tukey)		
								1 to 2	1 to 3	2 to 3
	M	SD	M	SD	M	SD	M (SD)	*p*	*p*	*p*
Customer incivility	2.09	0.57	2.86	0.63	3.03	0.72	2.64 (0.77)	***	***	0.20
Coworker incivility	1.38	0.41	1.55	0.48	1.90	0.58	1.61 (0.54)	0.06	***	***
Emotional exhaustion	2.20	0.74	2.27	0.75	3.58	0.83	2.71 (1.01)	0.83	***	***
Job performance	3.06	0.68	3.83	0.61	3.46	0.62	3.41 (0.71)	***	***	***
Turnover intention	2.80	0.88	2.43	0.70	3.84	0.75	3.06 (0.98)	**	***	***
LMX quality	3.70	0.60	4.34	0.58	3.12	0.66	3.67 (0.78)	***	***	***
Caring climate	3.71	0.76	3.96	0.65	2.76	0.76	3.44 (0.89)	**	***	***

Notes: *** *p* < 0.001, ** *p* < 0.05, *p* from post hoc ANOVA for mean comparison (two-tailed).

## Data Availability

Not applicable.
